# Holoprosencephaly

**DOI:** 10.1186/1750-1172-2-8

**Published:** 2007-02-02

**Authors:** Christèle Dubourg, Claude Bendavid, Laurent Pasquier, Catherine Henry, Sylvie Odent, Véronique David

**Affiliations:** 1 UMR 6061 CNRS, Institut de Génétique et Développement de Rennes, Université de Rennes1, IFR 140 GFAS, Faculté de Médecine, Rennes, 35000, France; 2 Laboratoire de Génétique Moléculaire et Hormonologie, Centre Hospitalier et Universitaire de Pontchaillou, Rennes, 35000, France; 3 Service de Génétique Médicale, Hôpital Sud, Rennes, 35000, France; 4 Laboratoire de Cytogénétique, Centre Hospitalier et Universitaire de Pontchaillou, Rennes, 35000, France

## Abstract

Holoprosencephaly (HPE) is a complex brain malformation resulting from incomplete cleavage of the prosencephalon, occurring between the 18th and the 28th day of gestation and affecting both the forebrain and the face. It is estimated to occur in 1/16,000 live births and 1/250 conceptuses. Three ranges of increasing severity are described: lobar, semi-lobar and alobar HPE. Another milder subtype of HPE called middle interhemispheric variant (MIHF) or syntelencephaly is also reported. In most of the cases, facial anomalies are observed in HPE, like cyclopia, proboscis, median or bilateral cleft lip/palate in severe forms, ocular hypotelorism or solitary median maxillary central incisor in minor forms. These latter midline defects can occur without the cerebral malformations and then are called microforms. Children with HPE have many medical problems: developmental delay and feeding difficulties, epilepsy, instability of temperature, heart rate and respiration. Endocrine disorders like diabetes insipidus, adrenal hypoplasia, hypogonadism, thyroid hypoplasia and growth hormone deficiency are frequent. To date, seven genes have been positively implicated in HPE: *Sonic hedgehog *(*SHH*), *ZIC2*, *SIX3*, *TGIF*, *PTCH*, *GLI2 *and *TDGF1*. A molecular diagnosis can be performed by gene sequencing and allele quantification for the four main genes *SHH*, *ZIC2*, *SIX3 *and *TGIF*. Major rearrangements of the subtelomeres can also be identified by multiplex ligation-dependent probe amplification (MLPA). Nevertheless, in about 70% of cases, the molecular basis of the disease remains unknown, suggesting the existence of several other candidate genes or environmental factors. Consequently, a "multiple-hit hypothesis" of genetic and/or environmental factors (like maternal diabetes) has been proposed to account for the extreme clinical variability. In a practical approach, prenatal diagnosis is based on ultrasound and magnetic resonance imaging (MRI) rather than on molecular diagnosis. Treatment is symptomatic and supportive, and requires a multidisciplinary management. Child outcome depends on the HPE severity and the medical and neurological complications associated. Severely affected children have a very poor prognosis. Mildly affected children may exhibit few symptoms and may live a normal life.

## Disease name and synonyms

Holoprosencephaly (HPE)

Midline cleft syndrome

DeMyer sequence

Isolated HPE (non syndromic, non chromosomic)

Familial HPE

Arhinencephaly

Cyclopia

## Definition

Holoprosencephaly (HPE, MIM 236100) is a complex human brain malformation resulting from incomplete cleavage of the prosencephalon into right and left hemispheres, occurring between the 18th and the 28th day of gestation. Three levels of increasing severity are described [1]: lobar HPE, where the right and left ventricles are separated, but with some continuity across the frontal cortex; semilobar HPE with a partial separation, and the most severe form, alobar HPE, with a single brain ventricle and no interhemispheric fissure. Another milder subtype of HPE called the middle interhemispheric variant (MIHF) or syntelencephaly, has now been recognized [2,3] (Table 1). There is a continuous spectrum of abnormal separation of the hemispheres rather than clearly distinct division into these three types of malformation [4]. The forebrain malformations are generally associated with facial anomalies, ranging from anophthalmia, cyclopia or proboscis in the most severe cases, to midline cleft lip, a simple hypotelorism or even no anomalies in the less severe HPE forms [5,6] (Table 2). The HPE phenotypic spectrum also encompasses microforms including facial midline anomalies with a normal brain. This wide spectrum can be observed within the same family [7].

**Table 1:** Anatomic classification of HPE -Different levels of severity [1] [6]

ALOBAR (complete)	Small single forebrain ventricle No interhemispheric division Absence of olfactory bulbs and tracts Absence of corpus callosum Non separation of deep gray nuclei
SEMI-LOBAR	Rudimentary cerebral lobes Incomplete interhemispheric division Absence or hypoplasia of olfactory bulbs and tracts Absence of corpus callosum Varying non separation of deep gray nuclei

LOBAR	Fully-developed cerebral lobes Distinct interhemispheric division Midline continuous frontal neocortex Absent, hypoplasic or normal corpus callosum Separation of deep gray nuclei

MIHF	Failure of separation of the posterior frontal and parietal lobes Callosal genu and splenium normally formed Absence of corpus callosum Hypothalamus and lentiform nuclei normally separated Heterotopic gray matter

**Table 2:** "The face often predicts the brain" [5, 110]. Parallelism of face and brain phenotypes generally observed in HPE.

Group	Morphology	HPE degree
(I) Cyclopia	Single or double eye Proboscis	Alobar
(II) Ethmocephaly	Distinct sockets Extreme ocular hypotelorism Proboscis	Alobar
(III) Cebocephaly	Ocular hypotelorism Proboscis, with single nostril	Alobar
(IVA)	Ocular hypotelorism Midline cleft lip Flat nose	Alobar or Semi-lobar
(IVB)	Ocular hypotelorism Midline cleft lip (complete or partial) Flat nose	Semi-lobar or lobar

HPE is a genetically heterogeneous anomaly and this phenotype is known to be part of different syndromes or chromosomal anomalies.

## Epidemiology

Holoprosencephaly is the most common forebrain developmental anomaly in humans with prevalence of 1/16,000 in live borns [8-11], an incidence as high as 1:250 in conceptuses [12], and a worldwide distribution. But considering the advances in neuroimaging with magnetic resonance imaging (MRI), children with less severe forms, like the recently described MIHF or lobar forms, who were undiagnosed, should be now identified leading to an increasing prevalence of the disease [13].

## Clinical description

As mentioned above, HPE is a complex brain malformation resulting from incomplete cleavage of the prosencephalon, affecting the forebrain. Therefore, clinical manifestations involve the central nervous system with possible facial dysmorphism and various complications [14]. Due to recent molecular data, the HPE phenotypic spectrum is very large ranging from severe cerebral malformations to a microform. This latter form can be defined by MRI normal brain, but with facial anomalies including ocular hypotelorism, midline cleft lip and/or flat nose.

Nowadays, most of severe HPE cases are detected by systematic ultrasound scan and MRI during pregnancy. This prenatal diagnosis often leads to termination of pregnancy, after genetic counseling, regarding to the severity of the malformations. Milder HPE cases or microforms are usually not available to prenatal diagnosis due to macroscopic normal brain. Nevertheless, these children show clinical spectrum of HPE described below.

In any case, it is important to establish whether the HPE is an isolated brain malformation or part of a syndrome with other systemic manifestations (Table 3)[15] to consider genetic counseling.

**Table 3:** Syndromic HPE with normal karyotype

**Syndromic HPE with normal karyotype**	
214800	CHARGE syndrome gene CHD7 8q12
146510	Pallister Hall gene GLI3 7p13
270400	Smith Lemli Opitz DHCR7 11q12-q13
180849	Rubinstein-Taybi gene CREBBP 16p11.3
249000	Meckel syndrome
264480	Pseudotrisomy 13
192430	Velo cardio facial Synd gene TBX1 22q11.2
300073	HPE and fetal akynesia, X linked?
300571	HPE, ectrodactyly, cleft lip/palate, X linked?
300373	Osteopathia striata with cranial sclerosis Xp11.4-p11.22
245552	Lambotte syndrome
184705	Steinfeld Syndrome
601357	HPE, amelia, facial cleft
600674	Microtia-anotia
236680	Hydrolethalus syndrome
206900	Microphtalmia syndromic
156810	MLRD association
202650	Dysgnathia complex
601370	Genoa Syndrome

### Anatomical description

The HPE brain malformation is an incomplete cleavage of the forebrain, with three classical levels of decreasing severity, alobar, semilobar and lobar, described in the definition and whose anatomic features are given in Table 1. This classic definition is not ambiguous, but problems are encountered at less severe ends of the phenotypic spectrum, which includes absent olfactory tracts and bulbs (arrhinencephaly), agenesis of the corpus callosum, hypopituitarism, single maxillary central incisor [16]. Less classical features were also described associated with HPE genes like hypoplasia of the pyriform aperture, ocular anomalies or anomalies of the extremities [7]. Simon *et al*. [4] established that the degree of deep gray nuclei non-separation parallels the degree of hemispheric non-separation.

In most of the cases, facial anomalies are observed in HPE. The severity of facial dysmorphism correlates with the cerebral anomalies in about 80% of the time (Table 2). These correlations were first given by DeMyer [5] then modified by several authors [9,17,18]. Less severe facial dysmorphism may include hypotelorism, lateral cleft lip, and/or iris coloboma [19], and sometimes normal faces can be observed. Some authors suggest following the classification modified by Elias *et al*. [18], in order to take into account patients with midline defects and a normal or sub-normal brain development, but this classification is essentially based on a genetic definition as it concerns most of the time members of a family who only share a mutation with the proband but do not present any brain MRI anomalies.

### Clinical evaluation in HPE children and complications

Children with HPE experience many medical problems that need to be detected:

#### Neurological signs

Developmental delay is present in all live born HPE patients, and seems in agreement with the severity of the brain malformation. Approximately half of the patients with HPE develop epilepsy. Hydrocephaly can occur during pre- or postnatal development. Many others signs like mental retardation, hypotonia, weakness, spasticity, dystonia and abnormal movements are described.

#### Craniofacial malformations

Microcephaly, hypotelorism or hypertelorism, midline or lateral cleft lip and/or palate, flat nose, iris coloboma, single maxillary central incisors and hypoplasia of the pyriform aperture are often noticed.

#### Endocrine disorders

Diabetes insipidus, growth hormone deficiency, adrenal hypoplasia, hypogonadism, or thyroid hypoplasia are very frequent in HPE because the midline malformation affects the development of the hypothalamus and the pituitary gland.

#### Oromotor dysfunction

Feeding and swallowing difficulties are frequent and generally correlate with the grade of HPE, leading to a requirement for gastrostomy tubes in about two thirds of patients with alobar or semilobar HPE [13]. These complications can be due to axial hypotonia, cleft lip and/or palate, gastro esophageal reflux, choking, slowness in eating and frequent pauses and often lead to failure to thrive.

#### Dysautonomic dysfunction

Instability of temperature, heart and/or breath rate.

### Prognosis

It is clear that neurodevelopmental outcome and mortality risk depend on the severity of HPE. Reports from the "Carter Centers for Brain Research in Holoprosencephaly and Related Malformations" (a national North American consortium funded by a not-for-profit foundation) on 83 children [20-22] and from Rennes (France) on 47 children [7] summarized some of the clinical problems and neurological disorders observed in HPE children. In these two series, less than half of the children had semilobar HPE; approximately 15% each had alobar, lobar or MIHF in the American series, while these forms represented respectively 17.8%, 27.3% and 17% in the French series.

It is generally reported that HPE children do not survive beyond early infancy [13,23], but this is essentially true for severe forms of HPE associated with severe craniofacial anomalies like cyclopia or ethmocephaly or chromosomal anomalies. A retrospective study of perinatal risk factors of 104 children living with HPE (Carter Center), performed by Stashinko *et al*. [24], showed that the mean age was 4 years and 15% of these children were between 10 and 19 years of age. In this case, children had normal vision and hearing, and could memorize in spite of their mental delay [11,25-27].

This underscores the importance of the Carter Neurocognitive Assessment, a new specific evaluation tool designed for children with HPE [28] and an accurate neuroradiologic classification of HPE [13] to help physicians and families in determining that outcome. Recently, a positive correlation was found out between the degree of non separation of the deep grey nuclei and expressive speech skills [20] but none regarding social awareness, visual attention and auditory comprehension [29].

However, the prognosis in Holoprosencephaly depends also on its etiology and is much poorer for those with cytogenetic abnormalities, with only 2% surviving beyond one year [11].

## Etiology

The etiology of HPE is very heterogeneous. First, this pathology can be caused by environmental or metabolic factors. The only formally recognized environmental factors are insulin-dependent diabetes mellitus (1% risk of HPE) [30] and maternal alcoholism with a risk that cumulates with smoking (RR 1.4) [31]. HPE in humans has also been noted in association with prenatal exposure to drugs (retinoic acid, cholesterol biosynthesis inhibitors [32] or to infections (cytomegalovirus [33,34], toxoplasma [35,36], rubella [36,37]). The OMIM classification shows that HPE can also be associated in about 25% of the cases with several defined multiple malformation syndromes with a normal karyotype, like Smith-Lemli-Opitz [38], Pallister Hall [39] or velo-cardio-facial syndrome [40] (Table 3).

HPE can be due to chromosomal abnormalities, with a higher prevalence observed in trisomy 13 (70%), trisomy 18 and triploidy. Analysis of recurrent chromosomal anomalies led to the identification of 12 candidate regions (named HPE1 to HPE12) on 11 chromosomes that may contain genes involved in HPE [41]. Large deletions involving HPE loci, like HPE3, are responsible for contiguous genes syndromes (*e.g*. del 7qter, Currarino syndrome).

Finally, HPE may be a solitary manifestation (neither chromosomal nor syndromic) and several genes are implicated in this isolated form of HPE.

### Genetics of isolated HPE

#### 1) Mode of transmission

This pathology was first described as autosomal dominant (MIM# 142945), with an incomplete penetrance and a variable expression [42]. The penetrance in autosomal dominant HPE is estimated to be 80%, but recent data also suggest a multigenic and multihit origin [43].

#### 2) Genes

**Figure 1:**
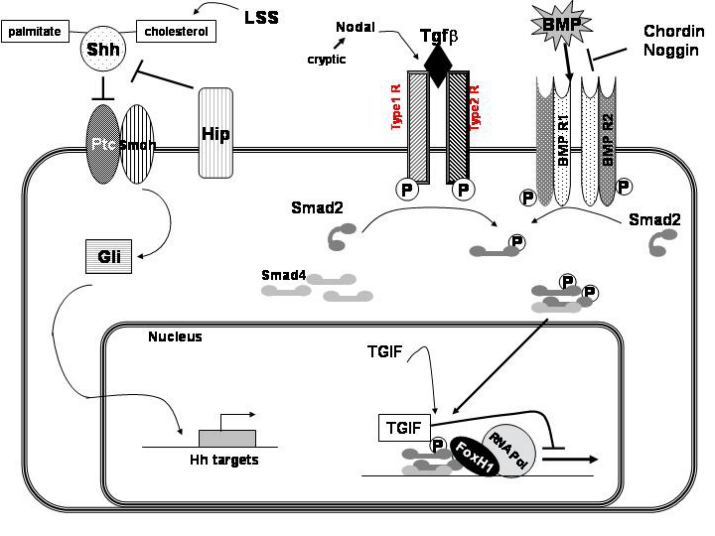
Signaling pathways of the main HPE genes or candidate genes. All the parts of these pathways are not necessarily in the same tissue or cell type.

The first genes positively implicated in HPE were identified from recurrent chromosomal rearrangements. Other genes were described by studying the SHH signaling pathway actors or the Nodal/TGFβ pathway, summarized in Figure 1.

***Sonic hedgehog*(*SHH*) **(MIM# 600725; 7q36; HPE3) [44,45]

*SHH *was isolated from the human critical region HPE3 on chromosome 7q36. It plays a critical role in early forebrain and central nervous system development. SHH is expressed in the human embryo in the notochord, the floorplate of the neural tube, the posterior limb buds and the gut [46]. The SHH protein is a secreted intercellular signaling molecule, which is synthesized as a precursor that undergoes autocatalytic cleavage into N-terminal domain (SHH-N) and C-terminal domain (SHH-C). During the autoprocessing reaction, a cholesterol moiety is covalently attached to the C-terminus of SHH-N [47]. Mouse and chicken embryos cultured *in vitro *and exposed to cyclopamine, an alkaloid blocker of Sonic hedgehog signaling, exhibit craniofacial anomalies and display phenotypes of HPE [48]. Homozygous *Shh *mutant mice present with cyclopia and often die during embryonic development, whereas the heterozygous mouse appears normal [49].

*SHH *is the major gene implicated in holoprosencephaly (12.7% of HPE cases: 50% of overall point mutations and 38% of overall large deletions) [50,51]. Some of the mutants were functionally characterized by studying the production of the active SHH-N fragment and the signaling activity in a reporter cell-based assay [52,53]. In families with *SHH *mutation, an incomplete penetrance and extremely wide phenotypic variability are observed [7]. Large deletions in 7q36, resulting in the loss of not only the *SHH *gene but also the *HLXB9 *gene have been observed in patients presenting HPE and Curarrino (sacral agenesia). Nevertheless, one study reported that only 50% of patients with del(7)(q36), including SHH, have HPE [43]. HPE can also be caused by apparently balanced translocations near the *SHH *locus, which might be as much as 200 kb away from the gene, due to position effect [45].

***ZIC2 ***(MIM# 603073; 13q32; HPE5) [54]

The second gene identified was *ZIC2*, which encodes a member of a transcription factor family that includes the Drosophila *odd-paired *gene (*opa*), and contains zinc finger DNA binding motifs similar to the GLI protein binding domains. ZIC2 plays an important role in neurulation. *Zic2 *knockdown mice show a strong holoprosencephaly phenotype in which the cerebral hemispheres are fused, and structures derived from the dorsal midline of forebrain are missing or reduced [55].

*ZIC2 *is the second HPE gene by order of involvement (9.2% of HPE cases: 31% of overall point mutations and 38% of overall large deletions). Small mutations identified include a significant part of complex mutations that are not just base substitutions but up to 30 base-pairs deletions, insertions or duplications, probably because of the gene sequence composition. Large deletions in 13q, including *ZIC2*, were narrowed by Comparative Genomic Hybridisation (CGH) array, which allows an inkling of correlations between the size of the rearrangement and the phenotype: one fetus with a typical HPE strictly limited to cerebral anomalies was found to have a deletion of 1, 5 Mb, while another fetus presenting an HPE in a polymalformative context carried a 30 Mb deletion.

Most of the time, alterations in *ZIC2 *correlate with a normal face and, in some cases, are associated with neural tube defects or syntelencephaly (MIHF). Spina bifida or anencephaly have been also described as being associated with HPE in the same patient or in the same family by several authors [7,8,56].

***SIX3 ***(MIM# 603714; 2p21; HPE2) [47]

*SIX3 *is an homeobox-containing gene which is homologous to the Drosophila *sine oculis *gene; it is involved in head midline and eye formation.

Mice with reduced levels of *Six3 *expression present a failure of forebrain and eye development [57].

Point mutations and large deletions in *SIX3 *represent 3% and 1% of HPE cases respectively. They are generally found in severe phenotypes. New phenotypes like atelencephaly – absence of telencephalon – or syntelencephaly – failure of separation of the posterior frontal and parietal right and left lobes [14,58] – may be associated; these phenotypes can be considered as a continuum of the HPE spectrum, based on embryological arguments and molecular results involving *SIX3 *[59].

***TGIF ***(MIM# 602630; 18p11.3; HPE4) [60].

TGIF (TG-interacting factor) is another homeodomain transcription factor that inhibits signaling through Nodal/Transforming Growth Factor beta (TGFβ) pathways by blocking the action of SMAD proteins. Moreover, TGIF is a repressor of retinoic acid regulated gene transcription (Figure 1).

Mutations in *TGIF *account for 1% of studied cases and large deletions for nearly 1%. These data suggest that *TGIF *alterations explain only a very small proportion of HPE cases, and TGIF involvement in HPE can be discussed since only 10% of patients carrying a 18p deletion (including the *TGIF *gene) present HPE [61,62] and mouse models with disrupted entire Tgif do not present an HPE phenotype [63]. However, recent studies report that heterozygous and homozygous deletions of the third exon of *Tgif *in mice can result in a defined spectrum of brain developmental defects including exencephaly, microcephaly, holoprosencephaly, and abnormalities in embryonic brain ventricle formation and cleavage [64]. The variable expression observed in families with one *TGIF *mutation makes genotype-phenotype correlations more difficult: both severe forms and labial/palate cleft are reported to be associated with mutations in this gene. Several hypotheses can be produced to explain these observations. First, it is possible that the *TGIF2 *gene (in 20q11.2-q12) provides redundancy for TGIF activity in the forebrain, even if Tgif2 does not have the same activity as Tgif in the neural tube in chick [65]. Additional studies will be required to elucidate the role of TGIF in prosencephalic neural development and HPE.

***PATCHED1 ***(MIM# 601309; 9q22) [66]

PATCHED-1 (PTCH) is the receptor for SHH, and normally acts to repress SHH signaling. This repression is relieved when SHH binds to PTCH. Four different mutations of the gene were described in five unrelated HPE-affected individuals. These mutations may affect the ability of PTCH to bind SHH, or perturb the intracellular interactions of PTCH with other proteins involved in SHH signaling. They could subsequently enhance the repressive activity of PTCH on the SHH pathway and so decrease SHH signaling.

***GLI2 ***(MIM# 165230; 2q14)

*GLI2 *is one of three vertebrate transcription factors implicated as obligatory mediators of SHH signal transduction. Mutations in this gene are preferentially involved in a distinctive phenotype (within the HPE spectrum) whose main features include defective anterior pituitary formation and pan-hypopituitarism [67], not necessarily with obvious abnormalities of the prosencephalon cleavage.

***TDGF1*/*CRIPTO ***(MIM# 187395; 3p21.31)

TDGF1 (*Teratocarcinoma Derived Growth Factor*), also called CRIPTO, is an EGF-CFC family member and an obligate co-receptor involved in NODAL signaling, a developmental program implicated in midline, forebrain, and left-right axis development in model organisms. CFC1 (CRYPTIC), another member of this family, has been demonstrated to be required for proper laterality development in humans.

Zebrafish rescue assays indicate a role for TDFG1 in midline and forebrain development. One mutation in *TDGF1 *(3p21.3) has been described in a patient with midline anomalies of the forebrain [68].

#### 3) Other candidate genes

Numerous candidate loci and candidate genes have been suggested for HPE (Table 4) (Figure 1). Among these genes, some were already investigated in HPE.

**Table 4:** Known genes and candidate genes for HPE

**LOCI and known HPE genes**		**Candidate genes:**	
236100	HPE1 21q22.3	**Investigated or under investigation**	
157170	HPE2 2p21 SIX3	600909	LSS 21q22,3 HPE1
142945	HPE3 7q36 SHH	605194	CFC1 2q21.1
142946	HPE4 18p11.3 TGIF	181590	SIL 1p32
609637	HPE5 13q32 ZIC2	605189	DKK1 10q11.2
605934	HPE6 2q37.1–q37.3	**Hypothetical**	
601309	HPE7 9q22.3 PTCH	602103	TMEM1 21q22.3
609408	HPE8 14q prox	600288	FOXA2 20p11
-	HPE9 20p13	607502	DISP1 1q42
-	HPE10 1q42-qter	609486	EAPP 14q13 HPE8
-	HPE11 5pter	609863	TECT1 12q24.1
-	HPE12 6q26-qter	603475	CHRD 3q27
600725	gene SHH 7q36	602991	NOG 17q22
602630	gene TGIF 18p11.3	600073	LPR2 2q24–q31
603073	gene ZIC2 13q32	601500	SMO 7q32.2
603714	gene SIX3 2p21	606178	HHIP 4q31.22
187395	gene TDGF1 3p23-p21	112262	BMP4 14q22.2
601309	gene PTCH 9q22	601265	NODAL 10q22.1
603621	gene FOXH1 8q24.3	601366	SMAD2/4 18q21
165230	gene GLI2 2q14	608707	CDO 11q23-q24
		605049	TWSG1 18p11.3

**FOX-H1 (FAST1) **(MIM# 603621; 8q24.3) is a co-transcriptional factor of SMAD2 and SMAD4, and intervenes in TGF-β, activin and nodal signaling pathways. These factors control early development in vertebrates and are vital for specification of the anterior-posterior axis. Recent studies on *Fox-H1 *null mutant mice showed that embryos failed to pattern the anterior-posterior axis, form the node, prechordal mesoderm, notochord and definitive endoderm. These mutants presented aberrant anterior head structures and abnormal cardiac development.

Mutations in *FOX-H1 *in HPE with cardiac malformations were only described in an abstract [69]; these variants would affect the DNA-binding domain or the SMAD interacting domain.

**The *lanosterol synthase *gene **(MIM# 600909; 21q22.3; HPE1) is located in the HPE1 critical region, where partial monosomy 21q was described in several HPE cases (.) [70-74]. Lanosterol synthase catalyzes a key step in the biosynthesis of cholesterol, essential in the maturation of the SHH protein. A previous study reported no mutation in a small cohort of 30 patients [75], but further investigations are needed before definitely ruling out this candidate.

**CFC1 (CRYPTIC) **(MIM# 605194; 2q21.1) is a member of the EGF-CFC family. EGF-CFC genes encode extra cellular proteins that act as essential cofactors for Nodal, a member of the transforming growth factor beta (TGF-beta) family, and play key roles in intercellular signaling pathways during vertebrate embryogenesis (germ-layer formation, anterior-posterior axis orientation and left-right axis specification) [76-80]. *CFC1 *could be considered as a candidate gene for HPE, even if loss-of-function mutations in human *CFC1 *(encoding the CRYPTIC protein) were identified only in patients with heterotaxic phenotypes (randomized organ positioning).

***SIL*(SCL-interrupting locus) **(MIM# 181590; 1p32), was considered by Karkera *et al*. [81] as a candidate HPE gene, as Sil mutants displayed prominent midline neural tube defects including delay or failure of neural tube closure and holoprosencephaly. *SIL *mutations were searched in a panel of HPE patients, but only several common polymorphisms were identified, suggesting that SIL is not a common factor in HPE pathogenesis in humans.

**The human *DKK1 *gene **(MIM# 605189; 10q11.2) was tested by Roessler *et al*. [82], as a result of the cyclopia observed in frogs lacking *dkk-1*. The *Dkk *gene family has been recently described as coding for secreted proteins of a novel class of proteins that act during development to bind and sequester members of the Bmp and Wnt families, establishing developmental zones free of the effects of these powerful morphogenes [83]. Search for mutations in a cohort of 100 HPE patients revealed four missense mutations with preserved activity in head induction assays in frogs, suggesting a limited role for this gene in HPE pathogenesis.

***TMEM1 *(Transmembrane protein 1) **(MIM# 602103; 21q22.3; HPE1) was designated EHOC-1 for epilepsy, holoprosencephaly candidate-1 as it was isolated from contigs of the candidate region HPE1. The protein is predicted to present multiple putative transmembrane domains that share partial homology with transmembrane proteins including sodium channel proteins [84].

***FOXA2 ***(MIM# 600288; 20p11); in zebrafish, the winged-helix transcription factor FoxA2 is involved in floor plate development and differentiation in conjunction with Cyclops (Nodal) signaling, and FoxA2 may be one component of the regulatory circuit controlling expression in the floor plate [85,86]. Moreover, Collignon *et al*. [87] showed that some mice manifest left-right axis malformations when doubly heterozygous for null mutations in FoxA2 and in Nodal.

***DISP1 ***(MIM# 607502; 1q42; HPE10) [88-91]. The encoded protein DISPATCHED (DISP1) presents twelve transmembrane domains and shares structural homology with the Patched Drosophila and vertebrates' gene in the form of a sterol-sensing domain. Disp functions to release cholesterol-anchored Hh and could be involved in multimeric Shh formation in membrane rafts [92].

***EAPP/C14ORF11 ***(MIM# 609486; 14q13; HPE8) was defined as a HPE potential candidate gene on chromosome 14 [93].

***TECT1/TECTONIC ***(MIM# 609863; 12q24.1). In mouse embryos, tectonic is expressed in regions that participate in hedgehog signaling. It is first expressed during gastrulation stages in the ventral node, and then in the gut endoderm, limb buds, notochord, somites, neural tube, and floor plate. Tectonic modulated hedgehog signaling downstream of smoothened and Rab23, and is required for maximal hedgehog activation [94].

***CHRD ***(MIM# 603475; 3q27) and NOG (MIM# 602991; 17q22). Chordin is a key developmental protein that dorsalizes early vertebrate embryonic tissues by binding to ventralizing TGF-beta-like bone morphogenetic proteins and sequestering them in latent complexes. Its dorsalizing effects are counteracted by BMP1 [75,95,96].

**The Noggin gene **was first discovered as an important factor in brain and nerve development. The secreted polypeptide noggin binds and inactivates members of the TGF-beta superfamily signaling proteins, such as bone morphogenetic protein 4 [97].

Some chordin/noggin double-null embryos present holoprosencephaly, with a single nasal pit, a cyclopic eye, and agnathia, and resembled embryos lacking sonic hedgehog. At embryonic day 12.5, double-mutant embryos were recovered with more severe phenotypes resembling aprosencephaly. In double-mutant embryos dissected at embryonic day 8.5, forebrain reduction was clearly evident. Chordin and noggin are not necessary for establishing the anterior visceral endoderm but are required for subsequent elaboration of anterior pattern [98]. BMP antagonists chordin and noggin compensate for each other during early mouse development. When both gene products are removed, antero-posterior, dorso-ventral, and left-right patterning are all affected.

**LPR2 **(Low Density Lipoprotein Receptor-Related Protein 2) (MIM# 600073; 2q24–q31) is also called glycoprotein 330 or megalin. Homozygous knockout mice manifest abnormalities in epithelial tissues [99]. In brain, impaired proliferation of neuroepithelium produces a holoprosencephalic syndrome, characterized by lack of olfactory bulbs, forebrain fusion, and a common ventricular system.

**SMOH **(MIM# 601500; 7q32.2)

Smoothened mediate with Patched the cellular response to the Hedgehog secreted protein signal; the binding of Sonic hedgehog to its receptor Patched prevents normal inhibition by PTCH of Smoothened (SMOH).

**HHIP **(Human Hedgehog Interacting protein) (MIM# 606178; 4q31.22) is involved in the attenuation of hedgehog signaling since ectopic expression of Hip in transgenic mice results in severe skeletal defects similar to those observed in Indian hedgehog mutants [100].

**BMP4 **(MIM# 112262; 14q22.2) is a vital regulatory molecule that functions throughout development in mesoderm induction, tooth development, limb formation, bone induction, and fracture repair. In expression studies in mouse, it was demonstrated that BMP4 activates the expression of Msx1, leading to incisor tooth development [101]. In chick embryos, the first signs of left-right asymmetry are detected in Hensen's node, essentially by left-sided Sonic hedgehog expression, and Bmp4 is necessary to maintain Shh asymmetry within the node [102].

**NODAL **(MIM# 601265; 10q22.1) is a member of the TGF-beta gene family and is expressed during mouse gastrulation [76]. Nodal has a left-sided expression pattern that is disrupted in mouse models of LR axis development [76]. Moreover, Collignon *et al*. [87] showed that some mice manifest left-right axis malformations when doubly heterozygous for null mutations in Nodal and in FoxA2.

***SMAD2/4 ***(MIM# 601366; 18q21). SMAD proteins mediate TGF-beta signaling to regulate cell growth and differentiation. TGF-beta induces activation and nuclear translocation of SMAD2 which forms complexes with SMAD4 and regulate transcription of target genes.

***CDO/CDON ***(MIM# 608707; 11q23-24) is another HPE candidate gene since mice lacking the transmembrane protein Cdo/Cdon, previously implicated in myogenesis, display HPE with strain-specific severity and without limb defects, modeling human HPE and implicating modifier genes as a cause of variability [103].

**The *Twisted gastrulation*(*TWSG 1*) gene **(MIM# 605049; 18p11.3; HPE4) is located near the *TGIF *gene. The TWSG protein is able to either enhance or inhibit signaling by the bone morphogenetic protein (BMP) subfamily of TGF-β type factors. All *Twsg1 *mutant mice, irrespective of genetic background, exhibit deletions of neural arches in the cervical vertebrae, and C57BL/6 ones present pronounced forebrain defects including rostral truncations, holoprosencephaly, cyclopia, agnathia [104].

#### 4) Debate on the multi-hit origin of HPE

HPE seems nowadays to be a multihit pathology, that may require two or more events involving several genes and/or environmental factors [43]. Indeed, some of mutations are found in a heterozygous state with a variable phenotypic penetrance in the same family, and double heterozygous mutations have already been identified (in *SHH *and in *ZIC2 *[44], in *SHH *and in *TGIF *[61], in *GLI2 *and in *ZIC2*, and two different missense mutations in *ZIC2 *in the same fetus. Moreover, a number of HPE cases consist of an association between several rearrangements detected by MLPA in different chromosomal regions and, in particular, a duplication associated with a deletion, like (7pdup;7qdel) or (8pdup;7qdel)[105]. The finding of such multiple rearrangements in a same patient suggests a balanced translocation in one of the normal parents and reinforces the multigenic and multihit origin. This multihit hypothesis can also offer explanation for the wide phenotypic spectrum described for a same mutation in a same family, the most severe form being due to the additive effect of several events. This conception can be illustrated by the finding of a *TGIF *nonsense mutation in a fetus presenting a semilobar HPE with cebocephaly, turricephaly, microcephaly and flat face detected by ultrasound, but also in her father who presented only mild signs like hypotelorism and lateral cleft lip, although this stop mutation was predicted to encode a truncated version of the TGIF protein missing two repression domains [106]. Animal models support this double heterozygosity hypothesis, since HPE is due to digenic inheritance in mouse models [43].

However the number of instances with double hits clearly identified and confirmed by functional studies remains relatively restricted to a few cases for the time being, unlike classic recessive disorders. HPE still behaves as an apparent autosomal dominant disorder with reduced penetrance and variable expression, associated with family-specific genetic alterations. These deleterious mutations give a susceptibility for developing HPE, upon which other factors (either genetic either environmental) modulate expression [107]. The genetic background would have an influence on the incidence of brain abnormalities, as it was described in the mutant mice with deletion of the third exon of Tgif, suggesting that genetic modifiers functionally interact with the mutant protein during embryonic brain development [64]. Whether or not these factors constitute a second hit remains to be debated.

## Diagnostic methods

Based on denaturing high performance liquid chromatography (DHPLC) and sequencing, point mutations are found in the four main HPE genes (*SHH*, *ZIC2*, *SIX3 *and *TGIF*) in about 20% of the cases (25% in living children and 15% in fetuses) [50]; mutations are distributed all over these genes. Mutations in the 5' regulatory regions of these genes are also suspected [107] and are under investigation. All kinds of mutations have been reported but missense mutations are more frequent than nonsense ones, which makes the interpretation complex. Whenever a missense mutation is found, it should be validated using a functional test suitable for the gene to assess its deleterious effect on the protein structure and/or function [52].

Recently, gene dosage methods like Quantitative Multiplex PCR of Short Fluorescent Fragments (QMPSF) or Multiplex Ligation Probe Amplification (MLPA) (MRC-Holland), were added to the molecular diagnosis process [108]. Large deletions in *SHH*, *ZIC2*, *SIX3 *and *TGIF *are found in 8% of the cases (4% in alive children and 12% in fetuses) [51,109]. While the proportion of point mutations is higher in living children than in fetuses, part of deletions is, on the contrary, higher in fetuses than in living patients, which may explain the more severe phenotype in fetuses leading to termination of pregnancy.

A systematic search for gains or losses in the subtelomeres by MLPA led to the identification of about 4.3% novel rearrangements involving known but also novel HPE loci. But, at the moment, this approach still concerns research field.

**Figure 2:**
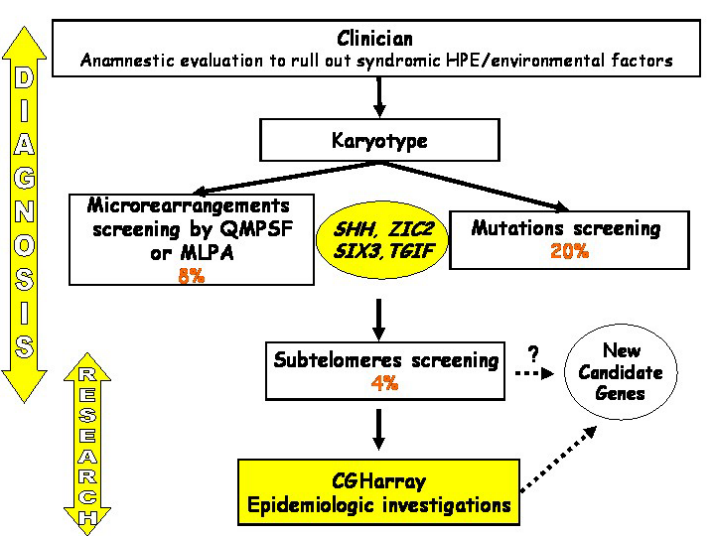
Molecular diagnosis of HPE.

These data lead to perform for each patient the successive molecular tests as described in the algorithm (Figure 2) as several hits are suspected to induce HPE.

## Management including treatment

The care of the child with HPE requires a multidisciplinary management. This aims to detect complications described above to avoid an added handicap and improve their quality of life.

Neurologic complications' management of a child with HPE is not specific and requires anticonvulsive, physical and occupational therapies, as for children with other brain malformations. It is important to consider endocrine disorders testing blood and urines samples and look after dysautonomic dysfunction. Surgery is needed to repair cleft lip and/or palate.

## Genetic counseling and prenatal diagnosis

The severity of HPE requires a genetic counseling, which is made difficult by the extreme phenotypic variability, the genetic heterogeneity, the multihit origin and a high risk of recurrence (13%) in apparently sporadic cases [42].

When a patient presents two associated subtelomeric rearrangements and, in particular, duplication associated with a deletion, it can be inherited from one of the parents carrying a balanced translocation, and so far, this could be a great help in the genetic counseling because of the high rate of recurrence.

On the contrary, when a mutation is identified in a patient but is not retrieved in his parents, the HPE onset involves a *de novo *mutation, and the recurrence rate cannot be calculated but is lower.

Prenatal ultrasound can detect the central nervous system and facial abnormalities of severe HPE as early as the first trimester, but is less sensitive for detection of milder forms of HPE. Fetal MRI will provide better characterization of the malformations in the third trimester of the pregnancy.

The greatest care must be taken for molecular prenatal diagnosis in HPE. Even if a mutation has been identified and seems to be transmitted with clinical manifestations in the family, another event, like a mutation in another gene (not yet identified) or an environmental factor, may be necessary to generate the holoprosencephaly phenotype [43]. In this case, molecular biology performed at the 10–12^th ^week of amenorrhea provides only an additional criterion with regard to prenatal ultrasound or MRI, which still takes precedence over molecular analysis. Nevertheless, the absence of a familial known mutation in the fetal DNA may be reassuring, before MRI imaging.

## Unresolved questions

When the search for alterations in the four main HPE genes and the analysis of subtelomeric regions are compiled, the combined rate reaches about 33%, so more than 65% of the cases remain unexplained, suggesting the involvement of many other genes in HPE. The research project should comprise the search for genomic rearrangements all over the genome, using a pangenomic approach like CGH array to identify further candidate genes. Moreover, epidemiologic investigations should be done to check off environmental factors that could act in coordination with genetic events to give rise to holoprosencephaly.
